# Association Between Ursodeoxycholic Acid and Clinical Outcomes in Patients With COVID-19 Infection: Population-Based Cohort Study

**DOI:** 10.2196/59274

**Published:** 2024-10-07

**Authors:** Hyunjun Lee, Min Gul Kim, Sang Woo Yeom, Sang Jae Noh, Cho Yun Jeong, Min Ji Kim, Min Gu Kang, Ji Hoon Ko, Su Cheol Park, Hyeok Tae Kweon, Sang Il Sim, Hyun Lee, Yeon Seok You, Jong Seung Kim

**Affiliations:** 1 Department of Otorhinolaryngology-Head and Neck Surgery, Jeonbuk National University Medical School, Jeonju, Republic of Korea Jeonju Republic of Korea; 2 Department of Pharmacology, Jeonbuk National University Medical School, Jeonju, Republic of Korea Jeonju Republic of Korea; 3 Department of Medical Informatics, Jeonbuk National University Medical School, Jeonju, Republic of Korea Jeonju Republic of Korea; 4 Research Institute of Clinical Medicine of Jeonbuk National University—Biomedical Research Institute of Jeonbuk National University Hospital, Jeonju, Republic of Korea Jeonju Republic of Korea; 5 Big Data Center, Jeonbuk National University Hospital Jeonju Republic of Korea; 6 Department of Internal Medicine, Hanyang University College of Medicine Seoul Republic of Korea; 7 Department of Medical Informatics Department of Otorhinolaryngology Jeonbuk National University School of Medicine and Hospital Jeonju Republic of Korea

**Keywords:** Covid 19, COVID-19, ursodeoxycholic acid, population-based cohort study, SARS-CoV-2, Coronavirus, pandemic, population-based, retrospective cohort study, propensity score, UDCA, public health, common data model, clinical, severity

## Abstract

**Background:**

Several studies have investigated the relationship between ursodeoxycholic acid (UDCA) and COVID-19 infection. However, complex and conflicting results have generated confusion in the application of these results.

**Objective:**

We aimed to investigate whether the association between UDCA and COVID-19 infection can also be demonstrated through the analysis of a large-scale cohort.

**Methods:**

This retrospective study used local and nationwide cohorts, namely, the Jeonbuk National University Hospital into the Observational Medical Outcomes Partnership common data model cohort (JBUH CDM) and the Korean National Health Insurance Service claim–based database (NHIS). We investigated UDCA intake and its relationship with COVID-19 susceptibility and severity using validated propensity score matching.

**Results:**

Regarding COVID-19 susceptibility, the adjusted hazard ratio (aHR) value of the UDCA intake was significantly lowered to 0.71 in the case of the JBUH CDM (95% CI 0.52-0.98) and was significantly lowered to 0.93 (95% CI 0.90-0.96) in the case of the NHIS. Regarding COVID-19 severity, the UDCA intake was found to be significantly lowered to 0.21 (95% CI 0.09-0.46) in the case of JBUH CDM. Furthermore, the aHR value was significantly lowered to 0.77 in the case of NHIS (95% CI 0.62-0.95).

**Conclusions:**

Using a large-scale local and nationwide cohort, we confirmed that UDCA intake was significantly associated with reductions in COVID-19 susceptibility and severity. These trends remained consistent regardless of the UDCA dosage. This suggests the potential of UDCA as a preventive and therapeutic agent for COVID-19 infection.

## Introduction

Ursodeoxycholic acid (UDCA) is an extensively used drug in patients with hepatocholestatic disease [1]. The representative functions of UDCA are protecting hepatocytes and cholangiocytes, lowering cholestasis, improving the function of the liver, and alleviating liver fibrosis [2]. In addition, UDCA is known to affect the pathophysiology of various diseases, such as respiratory inflammation, cystic fibrosis, Parkinson disease, and colon cancer, other than hepatocholestatic disease [3]. The physicochemical properties of UDCA known to date include anti-inflammatory, cytoprotective, antiapoptotic, and immune regulatory properties [4].

Recent research suggests that UDCA may reduce SARS-CoV-2 infections by downregulating angiotensin-converting enzyme 2 (ACE2) expression through the inhibition of farnesoid X receptor (FXR) signaling [5]. These findings were performed in vitro, in vivo, and ex vivo settings. Various experimental setups have drawn attention to the potential of UDCA in preventing COVID-19 infection. For instance, a multicenter retrospective cohort study on liver transplant recipients indicated that the use of UDCA significantly lowered the incidence of SARS-CoV-2 infection, though it did not affect the severity of the infection [6].

Conversely, other retrospective cohort studies with different designs and patient populations yielded varying results [7,8]. A single-center study using propensity score (PS) matching found that there was no UDCA impact on COVID-19 survival rates among hospitalized patients [7]. Similarly, a large multicenter study on patients with chronic liver disease found no significant differences in hospitalization rates or intensive care unit admissions between UDCA-treated and untreated groups [8].

These conflicting results highlight the complexities and challenges in establishing a clear relationship between UDCA and COVID-19 outcomes. In addition, the existing studies primarily focus on specific patient groups, leading to a gap in understanding UDCA’s effects on the general population’s COVID-19 infection risk and severity.

To address this gap and provide real-world evidence, additional research, including large-scale cohort analyses from diverse populations, is necessary. Herein, we investigate the association between UDCA and COVID-19 infection based on evidence obtained from a large-scale cohort.

## Methods

### Data Source

In this study, we used 2 data sets to determine how the intake of UDCA affects the occurrence and severity of COVID-19 infection. Specifically, we use herein a local cohort database that converts hospital data collected at Jeonbuk National University Hospital into the Observational Medical Outcomes Partnership common data model (JBUH CDM) and the Korean National Health Insurance Service claim–based database (NHIS). Both the NHIS and JBUH CDM data sets include inpatient and outpatient information.

The JBUH CDM cohort includes approximately 2 million people in Jeonbuk Province and contains data spanning approximately 16 years (from 2008 to 2023). The NHIS database is a nationwide cohort, which is a government-managed insurance company, wherein approximately 97% of the population of Korea is enrolled [9]. As health information data are collected, managed, and maintained by the National Health Insurance Corporation, a customized database is provided as needed for policy and academic research purposes. The data used in this study were randomly stratified, and information was extracted from 2015 to 2021 for 8,528,533 people from the NHIS database, considering age, gender, economic status, and underlying diseases. This data set was obtained by combining the COVID-19–related data managed by the Korea Disease Control and Prevention Agency. Owing to data security issues, applications to the NHIS can be made only after receiving approval from the institutional review board (IRB; data serial number: KDC-NHIS-2022-1-623).

### Patient Definition

It is necessary to set the patient group and target the disease through an appropriate operational definition based on the characteristics of the health insurance data. The disease definition uses the *ICD-10* (*International Statistical Classification of Diseases, Tenth* *Revision*) code; the COVID-19 patient definition uses the patient list provided by the Korean Disease Control and Prevention Agency; the drug-related definition uses the drug active ingredient code; and the treatment-related definition uses the health insurance fee code.

**Figure 1:**
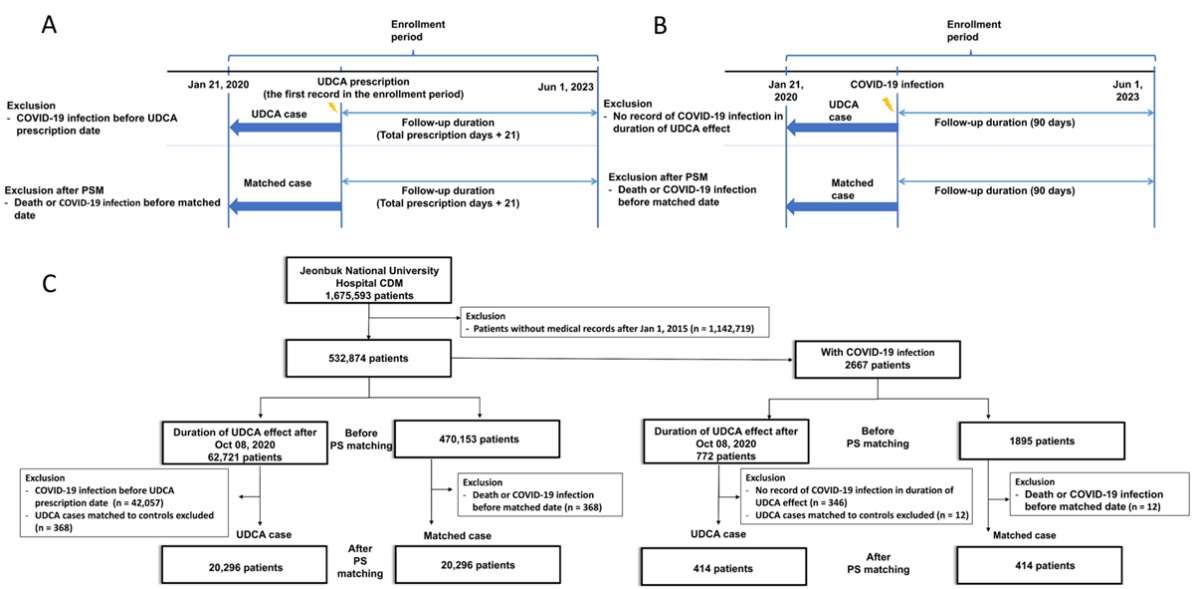
(A) Study design of COVID-19 susceptibility according to UDCA usage in the JBUH CDM database. (B) Study design of COVID-19 severity according to UDCA usage in the JBUH CDM database. (C) Flowchart of COVID-19 susceptibility and severity according to UDCA usage in JBUH CDM database. JBUH CDM: Jeonbuk National University Hospital Common Data Model; PS: propensity score; PSM: propensity score matching; UDCA: ursodeoxycholic acid.

**Figure 2:**
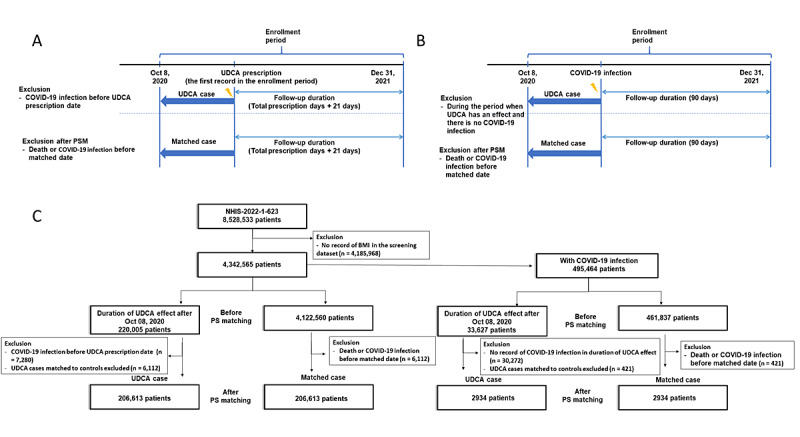
(A) Study design of COVID-19 susceptibility according to UDCA usage in the NHIS database. (B) Study design of COVID-19 severity according to UDCA usage in the NHIS database. (C) Flowchart of COVID-19 susceptibility and severity according to UDCA usage in the NHIS database case. NHIS: National Health Insurance Service; PS: propensity score; PSM: propensity score matching; UDCA: ursodeoxycholic acid.

The UDCA group was composed of those who had the prescription records of “2465” and “4480” in the first 4 digits of the drug active ingredient code. In addition, in the case of consecutive prescriptions, the difference between the first and last prescription dates was combined by adding the number of last prescription days. To determine whether this affects the incidence of COVID-19 infection in the UDCA group, the duration of the drug effect was defined as 21 days, and the follow-up period was defined by the total number of prescription days plus 21 days (from the first prescription date) [10-12]. In the JBUH CDM, patients with COVID-19 infection were available from January 21, 2020 (the date of the first COVID-19 outbreak in Korea), which is the first index date of our CDM study. In the JBUH CDM, the follow-up duration was set to June 1, 2023. In the NHIS data, patients with COVID-19 infection were included after October 8, 2020, owing to personal information protection issues. Therefore, in the NHIS data, the UDCA group, whose follow-up period was after October 8, 2020, was defined as the patient group of the NHIS study (Figures 1 and 2).

In cases of multiple COVID-19 infections, we considered the date of the first infection as the COVID-19 index date. The definition of patients with COVID-19 infection was a positive result from a real-time reverse transcriptase–polymerase chain reaction assay of nasal or pharyngeal swabs with a “U071” *ICD-10* code. This definition was applied in both the NHIS and JBUH CDM cases.

The inclusion criteria of UDCA administration for COVID-19 susceptibility or severity in the JBUH CDM and NHIS study were as follows: (1) age more than 19 years, and (2) UDCA users for at least 5 days.

The exclusion criteria for COVID-19 susceptibility in the JBUH CDM and NHIS studies were the following: (1) COVID-19 infection before the UDCA prescription date in the case of the UDCA group, (2) COVID-19 infection before the matched date in the case of the non-UDCA group, (3) deaths during the follow-up periods, (4) patients with no records after January 2015, and (5) patients who did not undergo the national health examination (conducted by the government) during the entire study period (Figures 1A and 1B and Figures 2A and 2B). The exclusion criteria for COVID-19 severity in the JBUH CDM and NHIS studies were as follows: (1) no COVID-19 infection in the case of the UDCA group during the period when UDCA had an effect, (2) COVID-19 infection before the matched date in the case of the non-UDCA group, (3) deaths during the follow-up periods, (4) patients with no records after January 2015, and (5) patients who did not undergo the national health examination (conducted by the government) during the entire study period. COVID-19 severity was defined when one of the following conditions occurred: (1) admission to an intensive care unit, (2) extracorporeal membrane oxygenation treatment, (3) use of a mechanical ventilator, or (4) death.

### Covariates

Five demographic variables, 6 underlying diseases, medication status affecting COVID-19 infection, COVID-19 vaccination, and the Charlson Comorbidity Index (CCI) were considered as the covariates. The five demographic variables were defined as follows: (1) sex: male or female; (2) age: 29, 30-59, 60-79, and more than 80 years; (3) economic status (based on insurance cost): top 30% (high), middle 40% (middle), and bottom 30% (low); (4) residential area: patients residing above the metropolitan city level in Korea are referred to as metro and those below are referred to as retro; and (5) and BMI: <25, 25-30, and >30.

To account for medications that could influence COVID-19 infection, we included the angiotensin receptor blocker (ARB), oral or intravenous corticosteroids (OCS), and inhaled corticosteroids (ICS). In addition, information on COVID-19 vaccinations—which could affect COVID-19 infections—was included as a covariate. Six underlying diseases recorded during the recruitment period (2015-2020) were defined based on the *ICD-10* code as follows: ascites (K7011, K7031, K7041, and K7151); autoimmune diseases (M32, M05, M06, K50, and K51); diabetes mellitus (DM) (E10, E11, E12, E13, E14, and 116 types of diabetes treatments); encephalopathy (G9380, G9381 A812, B220, E512, G934, I673, and I674); lung diseases (J44, J45, and J46), and nonalcoholic fatty liver disease (NAFLD) (K760). To ensure that no potential comorbid conditions were missed, we included an analysis using the CCI (including the following conditions: myocardial infarction, congestive heart failure, peripheral vascular disease, cerebrovascular disease [stroke], dementia, chronic pulmonary disease, connective tissue disease—rheumatic disease, peptic ulcer disease, mild liver disease, diabetes without complications, diabetes with complications, hemiplegia or paraplegia, renal disease [chronic kidney disease], any malignancies, including lymphoma and leukemia [excluding malignant neoplasm of skin], moderate or severe liver disease, metastatic solid tumor, and AIDS/HIV).

### Statistical Analysis

Primarily, Cox proportional hazards model was used to investigate the relationship between survival time and various factors. The Cox proportional hazards model is an extensively used statistical model in the field of survival analysis. This model, proposed by D R Cox in 1972, is used to analyze the hazard ratio (HR) of events over time in relation to various explanatory variables [13]. It is a type of regression analysis that incorporates the concept of time, allowing for the assessment of the impact of 1 or more variables on the occurrence of events (COVID-19 susceptibility or severity in our study).

We estimated the risk factors for the target and calculated the HR of the specific group compared with the control group. Unadjusted HR values were obtained using univariate analysis considering each variable and the target variable, and the adjusted HR (aHR) was obtained using multivariate analysis considering all other variables. A 2-sided *P* value of <.05 was considered statistically significant.

In all study designs, PS matching was performed for more validated comparisons between the UDCA and non-UDCA groups. PS matching is a statistical technique used to minimize selection bias in observational studies by matching individuals with similar PSs across different treatment groups. This method enhances the comparability of groups and strengthens the validity of causal inferences [14,15].

In PS matching, studies used a 1:1 ratio and a greedy algorithm because of the nature of large data. Confirmation of whether PS matching was successful was determined by the presence or absence of major imbalances at the baseline and standardized mean differences (SMDs) in each group. SMD values smaller than 0.2 indicated small levels of covariate imbalances; this means that the imbalance between covariates was overcome [9,16-19]. All statistical analyses were performed using SAS (version 9.4; SAS Institute Inc) and R (version 4.0.3; R Foundation for Statistical Computing).

### Ethical Considerations

This study was approved by the IRB of Jeonbuk National University Hospital (IRB number 2023-01-033). The informed consent was waived because all patient records were anonymized before use. No compensation was provided to participants as the study involved the use of anonymized big data.

## Results

### Study Population

In total, 1,675,593 patients were included in the JBUH CDM study. The JBUH CDM COVID-19 incidence study analyzed 532,874 patients classified into UDCA and matched cases. In the UDCA case, before PS matching, patients who were infected by COVID-19 infection before the UDCA prescription date (n=42,057) and matched to controls were excluded (n=368; Figure 1C). In the matched case, patients who died or were infected with COVID-19 infection before the matched date (n=368) were excluded. After PS matching, the number of patients in (each of) the UDCA and matched cases was 20,296 patients. In the JBUH CDM severity study, 2667 patients with COVID-19 infection were classified into UDCA and matched cases. Before PS matching, in the UDCA case, patients who had no record of COVID-19 infection in the duration of the UDCA effect (n=346) and matched to controls (n=12) were excluded. In the matched case, patients who died before the matching date (n=12) were excluded. After PS matching, the number of patients in (each of) the UDCA and matched cases was 414 patients. The procedure used regarding the population of the JBUH CDM study is shown in Figures 1A-1C.

The total population in the NHIS study included 8,528,533 patients. Patients with BMI records in the screening data set included 4,342,565 patients selected in the NHIS study. The NHIS COVID-19 incidence study analyzed 4,342,565 patients classified into UDCA and matched cases (Figure 2C). Before PS matching, in the UDCA case, patients who were infected by COVID-19 infection before the UDCA prescription date (n=7280) and matched to controls (n=6112) were excluded. In the matched case, patients who died or were infected with COVID-19 infection before the matched date (n=6112) were excluded. After PS matching, the number of patients in (each of) the UDCA and matched cases was 206,613 patients. In the NHIS severity study, 495,464 patients with COVID-19 infection were classified into the UDCA and matched cases. In the UDCA case, before PS matching, patients who had no record of COVID-19 infection in the duration of the UDCA effect (n=30,272) and those matched to controls (n=421) were excluded. In the matched case, patients who died before the matched date (n=421) were excluded. After PS matching, the number of patients in (each of) the UDCA and matched cases was 2934 patients. The procedure regarding the population of the NHIS study is shown in Figures 2A-2C.

### Validation of PS Matching

Tables S1 and S2 in Multimedia Appendix 1 show the frequencies and SMDs between UDCA and matched cases after PS matching in COVID-19 susceptibility and severity, respectively. All SMDs did not exceed the value of 0.2. There were no significant differences between the 2 groups (all SMDs <0.2).

### COVID-19 Susceptibility and Severity Analysis

**Figure 3:**
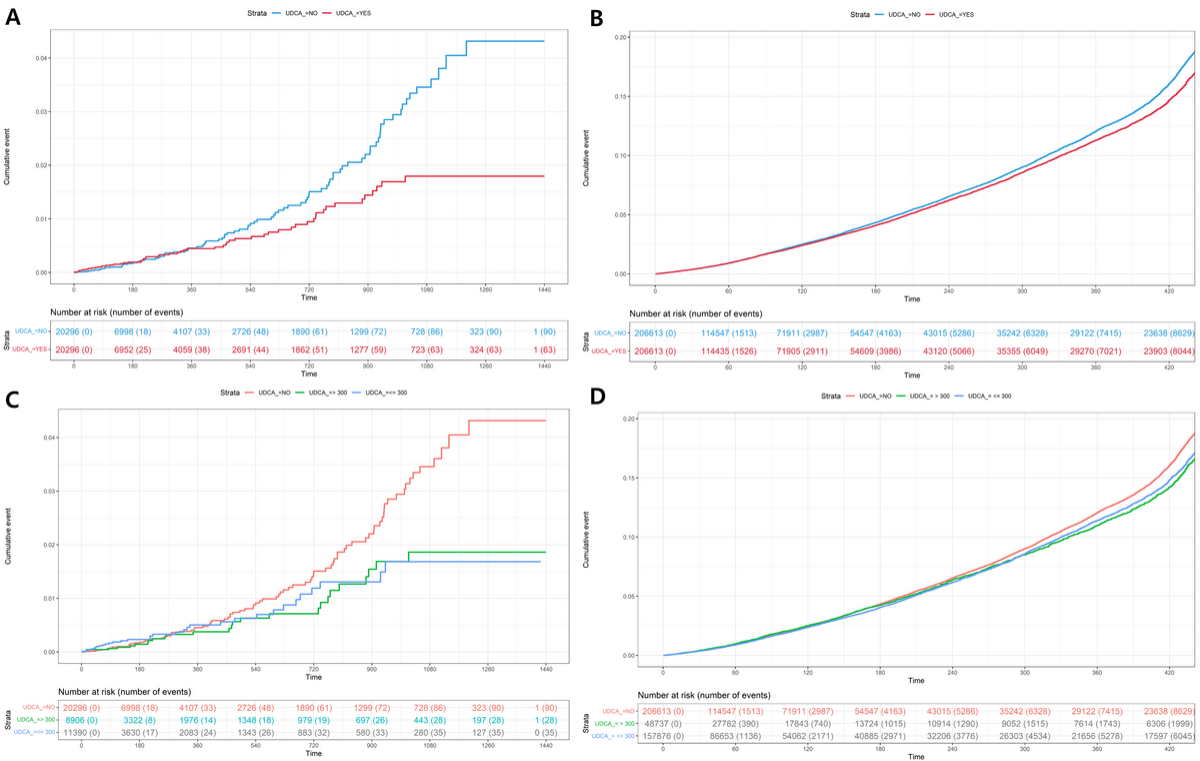
Cumulative incidence plots of (A) COVID-19 susceptibility according to UDCA usage in the Jeonbuk National University Hospital common data model (JBUH CDM) database case, (B) COVID-19 susceptibility according to UDCA usage in the National Health Insurance Service (NHIS) database case, (C) COVID-19 susceptibility according to UDCA dosage (≤300 mg; >300 mg) in the JBUH CDM database case, and (D) COVID-19 susceptibility according to the UDCA dosage (≤300 mg; >300 mg) in the NHIS database case. UDCA: ursodeoxycholic acid.

Table S3 in Multimedia Appendix 1 shows details of the analysis of COVID-19 susceptibility in JBUH CDM and NHIS data. In the JBUH CDM study, it was found that the aHR value was significantly lowered to 0.71 (95% CI 0.52-0.98; *P*=.03; Figure 3A). In the case of the UDCA group in the NHIS study, the aHR value for COVID-19 susceptibility was analyzed and was found to be significantly lowered to 0.93 (95% CI 0.90-0.96; *P*<.001; Figure 3B). In this study, we investigated the presence or absence of UDCA and the occurrence of COVID-19 infection according to the UDCA dose. In the JBUH CDM study, the aHR value of 0.64 (95% CI 0.42-0.98; *P*=.04) for doses >300 mg was significant, but the aHR value of 0.78 (95% CI 0.53-1.16; *P*=.20) for doses ≤300 mg was insignificant (Figures 3C and 5A). In the NHIS study, the aHR value of 0.94 (95% CI 0.91-0.97; *P*<.001) for doses ≤300 mg and the aHR value of 0.91 (95% CI 0.86-0.95; *P*=.001) for doses >300 mg were both significant (Figures 3D and 5B).

**Figure 4:**
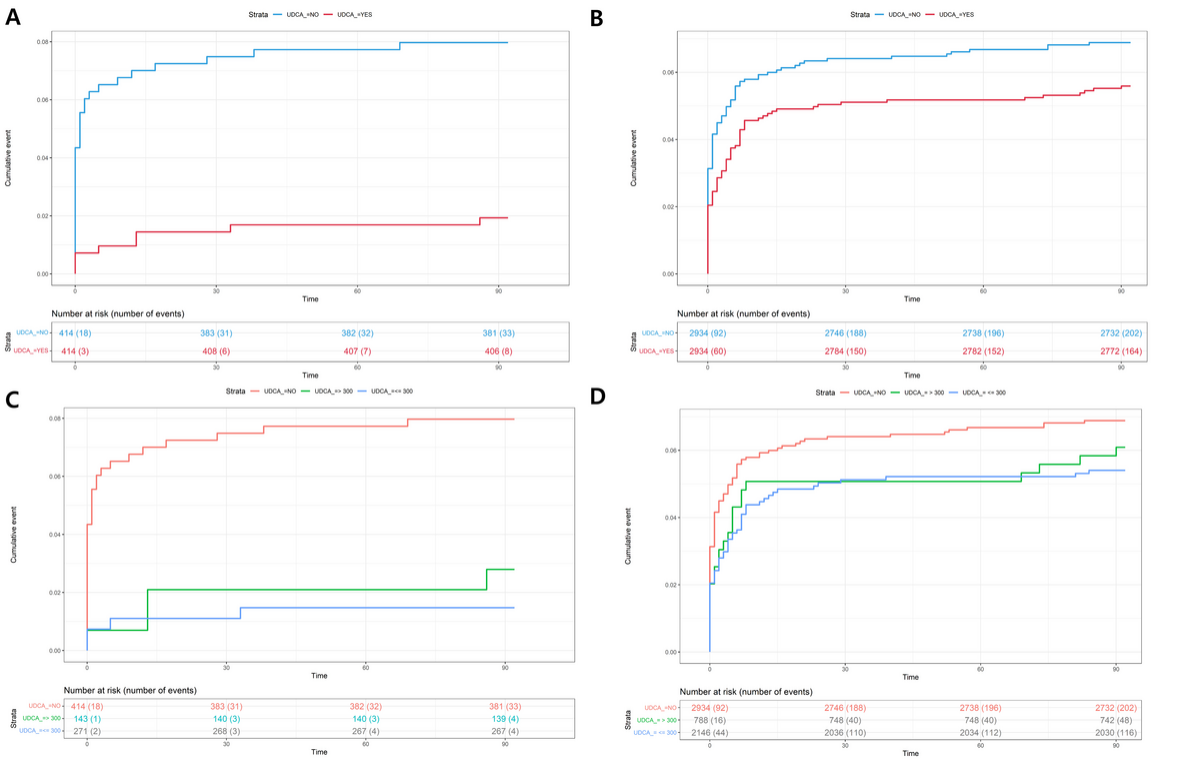
Cumulative incidence plots of (A) COVID-19 severity according to UDCA usage in the Jeonbuk National University Hospital common data model (JBUH CDM) database case, (B) COVID-19 severity according to UDCA usage in the National Health Insurance Service (NHIS) database case, (C) COVID-19 severity according to the UDCA dosage (≤300 mg, >300 mg) in the JBUH CDM database case, and (D) COVID-19 severity according to UDCA dosage (≤300 mg, >300 mg) in the NHIS database case. UDCA: ursodeoxycholic acid.

**Figure 5:**
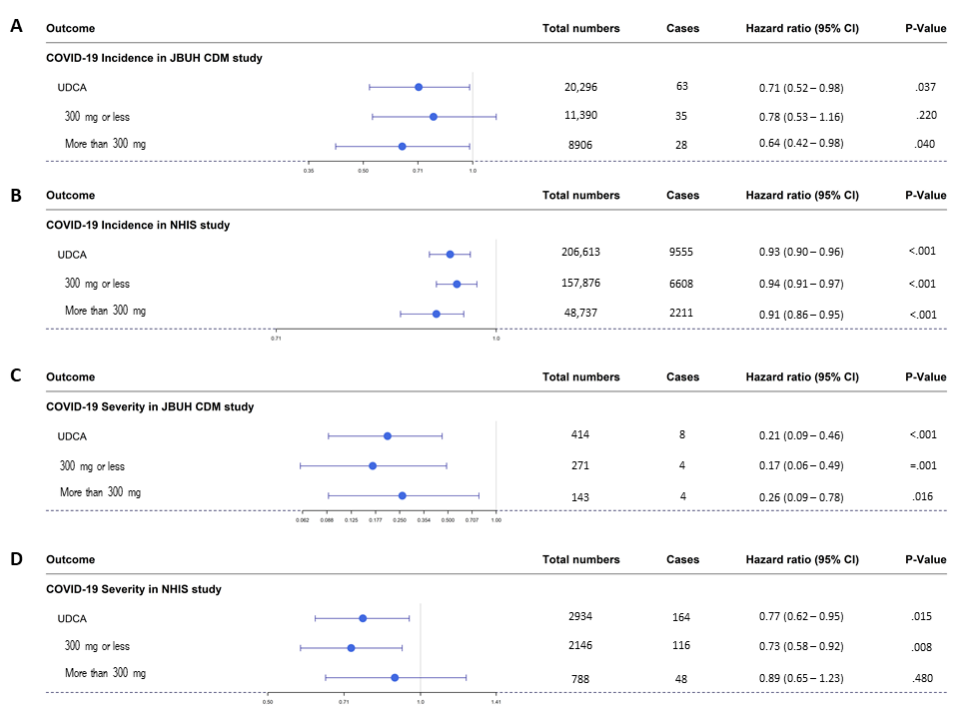
Forest plots of (A) COVID-19 susceptibility according to UDCA dosage (≤300 mg, >300 mg) in the JBUH CDM database case, (B) COVID-19 susceptibility according to UDCA dosage (≤300 mg, >300 mg) in the NHIS database case, (C) COVID-19 severity according to UDCA dosage (≤300 mg, >300 mg) in the JBUH CDM database case, and (D) COVID-19 severity according to UDCA dosage (≤300 mg, >300 mg) in the NHIS database case. JBUH CDM: Jeonbuk National University Hospital common data model; NHIS: National Health Insurance Service; UDCA: ursodeoxycholic acid.

Table S4 in Multimedia Appendix 1 shows details of the analysis of post–COVID-19 severity in the cases of the JBUH CDM and NHIS data. In the JBUH CDM study, it was found that the aHR value was significantly lowered to 0.21 (95% CI 0.09-0.46; *P*<.001; Figure 4A). In the case of the UDCA group in the NHIS study, the aHR value for severity was analyzed to be significantly lowered to 0.77 (95% CI 0.62-0.95; *P*=.02; Figure 4B). In the severity study, the analyzed results according to UDCA dose were also examined. In the JBUH CDM study, the aHR value of 0.17 (95% CI 0.06-0.49; *P*=.001) for doses ≤300 mg and the aHR value of 0.26 (95% CI 0.13-0.83; *P*<.001) for doses >300 mg were both significant (Figures 4C and 5C). In the NHIS study, the aHR value of 0.73 (95% CI 0.58-0.92; *P*=.01) for doses ≤300 mg was significant, but the aHR value of 0.89 (95% CI 0.65-1.23; *P*=.47) for doses >300 mg was insignificant (Figures 4D and 5D).

### Subgroup Analysis in COVID-19 Susceptibility

The results of Cox regression in the susceptibility study are specifically listed in Table S3 in Multimedia Appendix 1 and Figure 5. In the JBUH CDM susceptibility study, the incidence rate for COVID-19 infection in the group of those older than 80 years (>80) was 2.27 times higher (95% CI 1.41-3.66; *P*=.001) than that in the group of those aged 30 ≤ age < 60 (range 30-59) years. For underlying diseases, the incidence rate in the NAFLD group was 0.26 (95% CI 0.08-0.81; *P*=.03) times lower than in the non-NAFLD group. Gender (aHR 1.26, 95% CI 0.90-1.78; *P*=.18), age (<30 years, aHR 2.29, 95% CI 0.89-5.88; *P*=.09; 60 ≤ age < 80 years, aHR 1.20, 95% CI 0.81-1.77; *P*=.36), and underlying diseases such as ascites (aHR 0.90, 95% CI 0.39-2.06; *P*=.81), autoimmune diseases (aHR 1.51, 95% CI 0.55-4.13; *P*=.42), DM (aHR 1.15, 95% CI 0.77-1.72; *P*=.50), encephalopathy (aHR 1.09, 95% CI 0.15-7.93; *P*=.93), lung diseases (aHR 1.43, 95% CI 0.76-2.66; *P*=.27), and CCI (aHR 0.97, 95% CI 0.81-1.16; *P*=.74) also did not significantly affect the incidence rate. The use of ARB (aHR 1.35, 95% CI 0.90-2.02; *P*=.15), ICS (aHR 1.41, 95% CI 0.75-2.68; *P*=.29), and OCS (aHR 1.19, 95% CI 0.80-1.78; *P*=.39) showed no statistically significant differences in the incidence rate either.

In the NHIS susceptibility study, the incidence rate for COVID-19 infection in the male group was 0.81 (95% CI 0.79-0.84; *P*<.001) times lower than in the female group. Regarding age, the aHR values for the groups younger than 30 years (≤29), 60 ≤ age < 80 (60-79) years and older than 80 years (>80) were 1.33 (95% CI 1.20-1.47; *P*<.001), 0.83 (95% CI 0.81-0.86; *P*<.001), and 0.79 (95% CI 0.74-0.86; *P*<.001), respectively, compared with the group 30 ≤ age < 60 (range 30-59) years. Regarding economic status, the aHR values for the high group and the low group were each 0.92 (95% CI 0.89-0.95; *P*<.001) and 0.87 (95% CI 0.84-0.91; *P*<.001) compared with the middle group. Regarding residential areas, the incidence rate in the metro group was 1.49 times higher (95% CI 1.44-1.53; *P*<.001) than in the rural group. For BMI, the incidence rate in the group (BMI >30; severely obese patients) was 0.83 times lower (95% CI 0.79-0.87; *P*<.001) than in the normal group. For underlying diseases, the incidence rates in the ascites, DM, and NAFLD groups were 0.43 (95% CI 0.37-0.51; *P*<.001), 0.71 (95% CI 0.69-0.73; *P*<.001), and 0.77 times lower (95% CI 0.75-0.80; *P*<.001) than in each nondisease group. The incidence rate in the lung diseases group was 1.05 (95% CI 1.02-1.08; *P=*.001) times higher than in the non–lung diseases group. Single-point increases in the CCI score raised the incidence rate by 1.08 times (95% CI 1.08-1.09; *P*<.001). In the findings regarding the impact of medication use on COVID-19 susceptibility, the use of ICS and OCS increased the incidence rate by 1.12 times (95% CI 1.05-1.19; *P=*.001) and 3.03 times (95% CI 2.88-3.20; *P<*.001), respectively. Conversely, COVID-19 vaccination reduced the incidence rate by 0.16 times (95% CI 0.15-0.17; *P*<.001). Other underlying diseases, such as autoimmune diseases (aHR 0.98, 95% CI 0.94-1.02; *P*=.34), encephalopathy (aHR 0.98, 95% CI 0.80-1.20; *P*=.85), and the use of ARB (aHR 1.04, 95% CI 0.99-1.09; *P*=.12), did not significantly affect the incidence rate.

### Subgroup Severity Analysis

The results of Cox regression in the severity study are listed in Table S4 in Multimedia Appendix 1. In the JBUH CDM severity study, the incidence rate for severe COVID-19 infection in the ascites group was 5.42 times higher (95% CI 1.24-23.77; *P*=.03) than in the non–ascites group. However, sex (aHR 1.59, 95% CI 0.77-3.28; *P*=.21) and age (>80 years aHR 0.70, 95% CI 0.26-1.90; *P*=.48; 60 ≤ age < 80 years, aHR 1.26, 95% CI 0.60-2.64; *P*=.54) did not significantly impact the incidence rate. In addition, underlying diseases such as autoimmune diseases (aHR 1.18, 95% CI 0.16-8.77; *P*=.87), DM (aHR 0.82, 95% CI 0.36-1.85; *P*=.64), NAFLD (aHR 2.58, 95% CI 0.32-20.93; *P*=.37), encephalopathy (aHR 4.12, 95% CI 0.00-Infinite), lung diseases (aHR 2.43, 95% CI 0.88-6.76; *P*=.09), and CCI (aHR 1.06, 95% CI 0.72-1.56; *P*=.77) showed no statistically significant differences in the incidence rate. Also, the use of medications, such as ARB (aHR 0.96, 95% CI 0.42-2.24; *P*=.92), ICS (aHR 0.92, 95% CI 0.24-3.57; *P*=.90), and OCS (aHR 1.40, 95% CI 0.66-2.97; *P*=.38), also showed no statistically significant differences.

In the NHIS severity study, the incidence rate for severe COVID-19 infection in the male group was 1.65 times higher than in the female group (95% CI 1.29-2.11; *P*<.001). Regarding age, the aHRs values for the group 60 ≤ age < 80 (range 60-79) years and for patients older than 80 (≥80) years were 1.61 (95% CI 1.29-2.02; *P*<.001) and 3.56 (95% CI 2.51-5.04; *P*<.001), respectively, compared with that for the group 30 ≤ age < 60 (range 30-59) years. Regarding the economic status, the aHRs for the high and low groups were 1.29 (95% CI 1.01-1.65; *P*=.04) and 1.41 (95% CI 1.09-1.83; *P*=.01), respectively, compared with that of the middle group. For underlying diseases, the incidence rates in the ascites and DM groups were 2.46 (95% CI 1.43-4.22; *P*=.001) and 1.44 times higher (95% CI 1.15-1.81; *P*=.002) than in each disease group. Similarly, single-point increases in the CCI score raised the incidence rate by 1.07 times (95% CI 1.01-1.14; *P*=.02). In contrast, the vaccination group reduced the incidence rate of severe COVID-19 infection by 0.23 times (95% CI 0.14-0.39; *P*<.001) compared with the nonvaccinated group. However, other underlying diseases such as autoimmune diseases (aHR 0.93, 95% CI 0.70-1.22; *P*=.62), NAFLD (aHR 0.81, 95% CI 0.65-1.02; *P*=.06), encephalopathy (aHR 1.40, 95% CI 0.66-2.97; *P*=.38), and lung diseases (aHR 1.08, 95% CI 0.87-1.34; *P*=.49) showed no statistically significant differences in the incidence rate. Also, medication use, such as ARB (aHR 1.08, 95% CI 0.72-1.62; *P*=.71), ICS (aHR 1.36, 95% CI 0.75-2.46; *P*=.31), and OCS (aHR 0.70, 95% CI 0.47-1.06; *P*=.08), did not demonstrate statistically significant differences (Table S4 in Multimedia Appendix 1).

## Discussion

### Principal Findings

In our study, the use of UDCA significantly reduced the risk of COVID-19 infection caused by SARS-CoV-2 (JBUH CDM database: HR=0.71, 95% CI 0.52-0.98; *P*=.03; NHIS database: HR=0.93, 95% CI 0.90-0.96; *P*<.001), and it also significantly reduced the severity of COVID-19 infection (JBUH CDM database: HR=0.21, 95% CI 0.09-0.46; *P*<.001; NHIS database: HR=0.77, 95% CI 0.62-0.95; *P*=.02).

Brevini et al [5] suggested that taking UDCA can reduce SARS-CoV-2 infections. This study has reported that UDCA reduces FXR signaling, causing ACE2 to be downregulated, which can reduce SARS-CoV-2 infection. These results were confirmed in both in vitro and in vivo studies. These findings were also confirmed in an ex vivo study by an independent validation cohort.

Three UDCA properties led to this result. First, UDCA is known to reduce proinflammatory cytokines such as interleukin (IL)-1, IL-2, IL-6, and tumor necrosis factor α [20]. Antioxidant and antiapoptotic UDCA effects also aid this anti-inflammatory effect [4]. Taken together, UDCA reduces proinflammatory cytokines, preventing the progression of diseases to acute respiratory distress syndrome by suppressing the cytokine storm syndrome [4,21]. Second, UDCA prevents SARS-CoV-2 from binding to the receptor-binding domain of the spike protein of ACE2 [22,23]. The last one, identified in the rat model experiment, UDCA adjusted the ALX/cAMP/PI3K pathway and increased the clearance of the alveolar fluid of rats with acute respiratory distress syndrome [24,25].

As the COVID-19 pandemic progresses, strict controls for SARS-CoV-2 infection are being lifted in many countries worldwide. The principle of treatment is shifting from hospitalization to home-based care. While COVID-19 vaccines had some level of effectiveness, their efficacy decreased as new variant viruses such as omicron emerged [26,27]. Medications, such as remdesivir and nirmatrelvir or ritonavir, have various side effects, such as hepatotoxicity, limiting their use for the general population [28,29]. In addition, monoclonal antibodies, such as tixagevimab, cilgavimab (Evusheld), and bebtelovimab, have limitations owing to their high costs and the need for intravenous injections [30]. Consequently, there is a growing need for over-the-counter, low side effects, and oral administration of drugs for SARS-CoV-2 treatment and prevention. In this context, research on how UDCA reduces the susceptibility to and severity of SARS-CoV-2 infection has gained attention in various studies.

However, since the publication of relevant papers, such as that by Brevini et al [5], many studies on UDCA and COVID-19 infection have subsequently emerged that reported conflicting findings.

The recent research finding that UDCA intake does not prevent COVID-19 infection is limited owing to the participation of a small number of children (n=280), and questionnaire studies could be affected by voluntary response bias [31]. Another recent study that showed that UDCA did not improve the prognosis of SARS-CoV-2 infection in hospitalized patients also had the following limitations: (1) a very small sample size (57 out of 3847 patients), (2) the inclusion of only hospitalized patients, and (3) the fact that validated statistical methods, such as PS matching, were not used [32].

In our study, COVID-19 susceptibility was found to decrease by 0.71 times in CDM and 0.93 times in NHIS. This trend was even more pronounced in the subgroup with NAFLD in our study (CDM: 0.26 times, NHIS: 0.77 times). These effects are quite interesting, as UDCA appears to alleviate NAFLD by inhibiting apoptosis and increasing autophagy via activating the AMP-activated protein kinase [33]. UDCA improves NAFLD by reducing liver inflammation through the modulation of the FXR and other nuclear receptors. This modulation changes the composition of the gut microbiota, which is critical for bile acid metabolism, leading to decreased bile acid toxicity and enhanced anti-inflammatory effects, ultimately reducing COVID-19 susceptibility in patients with NAFLD [34,35]. The fact that COVID-19 susceptibility is reduced when UDCA is used in the subgroup with NAFLD is noteworthy.

In the analysis of drugs affecting COVID-19 infection, ARBs were found to have no significant impact. However, ICS (1.41 [CDM], 1.12 [NHIS]) and OCS (1.19 [CDM], 3.03 [NHIS]) generally increased COVID-19 susceptibility. This suggests that the immunosuppressive effects of steroids cannot be mitigated by UDCA.

Regarding COVID-19 severity, UDCA intake was found to reduce it by 0.21 times in CDM and 0.77 times in NHIS. These trends were consistently higher in subgroups with ascites, which were expected to have severe liver disease (CDM: 5.42, NHIS: 2.46). These results align with the findings of the study by John et al [30], where COVID-19 severity was 0.45 times lower in a PS-matched group of 1607 UDCA users with liver cirrhosis. This study is similar to our research in that it compared a relatively large number of UDCA users with PS-matched non-UDCA users [30].

Although various studies have been conducted, most have relied on small cohort studies with limited control over various variables. In this context, our study had the following strengths. First, the effects of UDCA on SARS-CoV-2 infection were demonstrated in a larger cohort than in other studies. In addition, while other studies have examined the effect of UDCA use on the susceptibility to and severity of SARS-CoV-2 infection in patients with specific liver and cholestatic diseases, our study examined the impact of general UDCA use on SARS-CoV-2 infection without targeting specific patient groups. We found that it was effective in the general population but not in treating specific disease groups. Therefore, the association between UDCA and SARS-CoV-2 infection in the general public could be predicted more accurately than in other studies. Another advantage is that, unlike other studies that could not use PS matching owing to the small sample size, our study was able to show the effect of UDCA even after the exclusion of various variables through PS matching. In addition, a cohort study using 2 different patient groups was used to study the effects of UDCA in the Jeonbuk region (JBUH CDM: local cohort) and the entire country (NHIS: nationwide cohort). The same findings were obtained in the 2 cohorts that showed that UDCA lowered the susceptibility and severity of SARS-CoV-2 infection. Furthermore, our study differed from others in that we investigated the dosage of UDCA and its relationship with the susceptibility and severity of COVID-19 infection. We compared the effects of the administration of UDCA doses ≤300 mg and >300 mg, whereby the former dose category reduced only the COVID-19 susceptibility by 0.94 times in the NHIS study, while the latter dose category reduced susceptibility by 0.64 times in the JBUH CDM study and by 0.91 times in the NHIS study. In addition, doses ≤300 mg reduced COVID-19 severity by 0.17 times in the JBUH CDM study and by 0.73 times in the NHIS study, whereas doses >300 mg reduced severity only by 0.26 times in the JBUH CDM study. This aligns with the results of the previous study, where UDCA consumption at 5 mg/kg body weight reduced susceptibility to COVID-19 infections by 0.83 times and led to reduced moderate COVID-19 infection rates by 0.77 times [30]. In terms of COVID-19 severity, the low-dose UDCA group showed significant results across the JBUH CDM and NHIS study, whereas the high-dose UDCA group had some nonsignificant outcomes. This discrepancy is attributed to the high-dose group having more severe comorbidities, which may have hindered the effectiveness of UDCA alone in reducing COVID-19 severity. As a result, our study was able to predict the relationship between UDCA and SARS-CoV-2 infection more accurately.

Regarding the COVID-19 vaccine, it reduced COVID-19 susceptibility and severity by 0.16 and 0.23 times, respectively. This is significantly lower than the original effects of UDCA, which reduced these factors by 0.93 and 0.77 times. COVID-19 vaccines inherently train the immune system to recognize and fight against SARS-CoV-2, while UDCA may provide an additional layer of protection by reducing the ability of SARS-CoV-2 to enter the host cells [5,36]. The combined use of UDCA and COVID-19 vaccines could potentially offer enhanced protection; however, additional research is needed to understand this relationship in depth.

To account for potential missing comorbidities, we included the CCI in our analysis. The results showed that with single-point increases in the CCI, the likelihood of COVID-19 infection increased by 1.08 times, and the likelihood of progressing to severe COVID-19 infection increased by 1.07 times in relation to UDCA and COVID-19 susceptibility or severity. This suggests that although UDCA can reduce COVID-19 susceptibility and severity, its effectiveness may be diminished by the presence of comorbidities.

However, our study has the following limitations. As a retrospective design study, the research design itself had shortcomings. Additional future research on the relationship between UDCA and SARS-CoV-2 infection through randomized controlled trials will enrich our results.

### Conclusions

Large-scale local and nationwide cohorts confirmed that UDCA intake significantly reduced COVID-19 susceptibility and severity. This suggests the potential of UDCA as a preventive and therapeutic agent against COVID-19 infection.

## Appendix

Multimedia Appendix 1 Standardized mean differences and incidence rates with hazard ratios from the National Health Insurance Service claim-based database and Jeonbuk National University Hospital into the Observational Medical Outcomes Partnership common data model COVID-19 susceptibility and severity studies.

## Abbreviations

ACE2: angiotensin-converting enzyme 2
aHR: adjusted hazard ratio
ARB: angiotensin receptor blocker
CCI: Charlson Comorbidity Index
CDM: common data model
DM: diabetes mellitus
FXR: farnesoid X receptor
HR: hazard ratio
ICD-10: International Statistical Classification of Diseases, Tenth Revision
ICS: inhaled corticosteroids
IL: interleukin
IRB: institutional review board
JBUH: Jeonbuk National University Hospital
NAFLD: nonalcoholic fatty liver disease
NHIS: National Health Insurance Service
OCS: oral or intravenous corticosteroids
PS: propensity score
SMD: standardized mean difference
UDCA: ursodeoxycholic acid
