# Supplementary material for: Association Between Ursodeoxycholic Acid and Clinical Outcomes in Patients With COVID-19 Infection: Population-Based Cohort Study
Source: JMIR Public Health Surveill. 2024 Oct 7;10:e59274. doi: 10.2196/59274 (PMC11494262; doi:10.2196/59274)
Supplement: Multimedia Appendix 1 [file publichealth_v10i1e59274_app1.docx]

**Table S1**. SMDs between the UDCA and matched cases after propensity score matching in the NHIS and JBUH CDM COVID-19 susceptibility study.

|  | **JBUH CDM** | | | **NHIS** | | |
| --- | --- | --- | --- | --- | --- | --- |
| Variable | UDCA case (n=20,296) (%) | Matched case (n=20,296) (%) | SMD | UDCA case (n=206,613) (%) | Matched case (n=206,613) (%) | SMD |
| Sex |  |  | 0.000 |  |  | 0.019 |
| Male | 11,889 (58.6) | 11,893 (58.6) |  | 131,721 (63.8) | 133,617 (64.7) |  |
| Female | 8,407 (41.4) | 8,403 (41.4) |  | 74,892 (36.3) | 72,996 (35.3) |  |
| Age |  |  | 0.008 |  |  | 0.082 |
| ~ 29 | 1,109 (5.5) | 1,108 (5.5) |  | 5,351 (2.6) | 6,145 (2.8) |  |
| 30 ~ 59 | 7,360 (36.3) | 7,316 (36.1) |  | 105,633 (51.1) | 98,579 (47.7) |  |
| 60 ~ 79 | 9,429 (46.5) | 9,427 (46.5) |  | 88,518 (42.8) | 92,530 (44.8) |  |
| 80 ~ | 2,398 (11.8) | 2,445 (12.1) |  | 7,111 (3.4) | 9,359 (4.5) |  |
| Economic status |  |  | - |  |  | 0.02 |
| High | - | - |  | 66,453 (32.2) | 68,248 (33.0) |  |
| Middle | - | - |  | 98,933 (47.9) | 98,245 (47.6) |  |
| Low | - | - |  | 41,227 (19.9) | 40,120 (19.4) |  |
| Residential area |  |  | - |  |  | 0.01 |
| Metro | - | - |  | 89,437 (43.3) | 88,425 (42.8) |  |
| Rural | - | - |  | 117,176 (56.7) | 118,188 (57.2) |  |
| BMI |  |  | - |  |  | 0.032 |
| Normal | - | - |  | 94,302 (45.6) | 92,760 (44.9) |  |
| 25 ~ 30 | - | - |  | 86,143 (41.7) | 89,132 (43.1) |  |
| Over 30 | - | - |  | 26,168 (12.7) | 24,721 (12.0) |  |
| Ascites |  |  | 0.009 |  |  | 0.031 |
| Yes | 377 (1.9) | 352 (1.7) |  | 2,734 (1.3) | 2,055 (1.0) |  |
| No | 19,919 (98.1) | 19,944 (98.3) |  | 203,879 (98.7) | 204,558 (99.0) |  |
| Autoimmune  diseases |  |  | 0.004 |  |  | 0.031 |
| Yes | 326 (1.6) | 316 (1.6) |  | 36,460 (17.7) | 34,089 (16.5) |  |
| No | 19,970 (98.4) | 19,980 (98.4) |  | 170,153 (82.4) | 172,524 (83.5) |  |
| DM |  |  | 0.003 |  |  | 0.009 |
| Yes | 2,212 (10.9) | 2,195 (10.8) |  | 80,809 (39.1) | 79,925 (38.7) |  |
| No | 18,084 (89.1) | 18,101 (89.2) |  | 125,804 (60.9) | 126,688 (61.3) |  |
| NAFLD |  |  | 0.002 |  |  | 0.019 |
| Yes | 703 (3.5) | 710 (3.5) |  | 64,061 (31.0) | 62,223 (30.1) |  |
| No | 19,593 (96.5) | 19,586 (96.5) |  | 142,552 (69.0) | 144,390 (69.9) |  |
| Encephalopathy |  |  | 0.014 |  |  | 0.022 |
| Yes | 67 (0.3) | 52 (0.3) |  | 205,358 (99.4) | 205,693 (99.6) |  |
| No | 20,229 (99.7) | 20,244 (99.7) |  | 1,255 (0.6) | 920 (0.5) |  |
| Lung diseases |  |  | 0.004 |  |  | 0.007 |
| Yes | 709 (3.5) | 694 (3.4) |  | 77,848 (37.7) | 77,181 (37.4) |  |
| No | 19,587 (96.5) | 19,602 (96.6) |  | 128,765 (62.3) | 129,432 (62.6) |  |
| ARB |  |  | 0.053 |  |  | 0.085 |
| Yes | 3,329 (16.4) | 2,938 (14.5) |  | 40,680 (19.7) | 33,912 (16.4) |  |
| No | 16,967 (83.6) | 17,358 (85.5) |  | 165,933 (80.3) | 172,701 (83.6) |  |
| ICS  Yes  No | 1,117 (5.5)  19,179 (94.5) | 1,148 (5.7)  19,148 (94.3) | 0.006 | 10,947 (5.3)  195,666 (94.7) | 10,102 (4.9)  196,511 (95.1) | 0.019 |
| OCS  Yes  NO | 3,167 (15.6)  17,129 (84.4) | 3,337 (16.4)  16,959 (83.6) | 0.023 | 88,219 (42.7)  118,394 (57.3) | 82,546 (40.0)  124,067 (60.0) | 0.055 |
| Vaccine  Yes  No |  |  |  | 102,893 (49.8)  103,720 (50.2) | 101,143 (49.0)  105,470(51.0) | 0.017 |
| CCI (mean, SD) | 0.76 (1.14) | 0.62 (1.01) | 0.133 | 2.25 (2.94) | 1.77 (2.61) | 0.173 |

Abbreviations: NHIS, the national health insurance service; JBUH, Jeonbuk national university hospital; CDM, common data model; UDCA, ursodeoxycholic acid; SMD, standardized mean difference; DM, diabetes mellitus; NAFLD, nonalcoholic fatty liver disease; ARB, Angiotensin receptor blocker; ICS; Inhaled corticosteroid; OCS, Oral corticosteroid; CCI, Charlson comorbidity index; SD, standard deviation.

**Table S2.** SMDs between the UDCA and matched cases after propensity score matching in the NHIS and JBUH CDM COVID-19 severity study.

|  | **JBUH CDM** | | | **NHIS** | | |
| --- | --- | --- | --- | --- | --- | --- |
| Variable | UDCA case (n=414) (%) | Matched case (n=414) (%) | SMD | UDCA case (n=2,934) (%) | Matched case (n=2,934) (%) | SMD |
| Sex |  |  | 0.045 |  |  | 0.004 |
| Male | 260 (62.8) | 269 (65.0) |  | 1,978 (67.4) | 1,984 (67.6) |  |
| Female | 154 (37.2) | 145 (35.0) |  | 956 (32.6) | 950 (32.4) |  |
| Age |  |  | 0.041 |  |  | 0.045 |
| ~ 29 | 20 (4.8) | 20 (4.8) |  | 66 (2.3) | 70 (2.4) |  |
| 30 ~ 59 | 117 (28.2) | 114 (27.5) |  | 1,578 (53.8) | 1,522 (51.9) |  |
| 60 ~ 79 | 190 (45.9) | 186 (44.9) |  | 1,154 (39.3) | 1,186 (40.4) |  |
| 80 ~ | 87 (21.1) | 94 (22.7) |  | 136 (4.6) | 156 (5.3) |  |
| Economic status |  |  | - |  |  | 0.016 |
| High | - | - |  | 952 (32.5) | 964 (32.9) |  |
| Middle | - | - |  | 1,290 (43.9) | 1,298 (44.2) |  |
| Low | - | - |  | 692 (23.6) | 672 (22.9) |  |
| Residential area |  |  | - |  |  | 0.022 |
| Metro | - | - |  | 1,530 (52.2) | 1,498 (51.1) |  |
| Rural | - | - |  | 1,404 (47.8) | 1,436 (48.9) |  |
| Ascites |  |  | 0.150 |  |  | 0.216 |
| Yes | 11 (2.7) | 3 (0.7) |  | 78 (2.7) | 4 (0.1) |  |
| No | 403 (97.3) | 411 (99.3) |  | 2,856 (97.3) | 2,930 (99.9) |  |
| Autoimmune  diseases |  |  | 0.031 |  |  | 0.061 |
| Yes | 11 (2.7) | 9 (2.2) |  | 546 (18.6) | 478 (16.3) |  |
| No | 403 (97.3) | 405 (97.8) |  | 2,388 (81.4) | 2,456 (83.7) |  |
| DM |  |  | 0.013 |  |  | 0.012 |
| Yes | 70 (16.9) | 72 (17.4) |  | 1,658 (56.5) | 1,676 (57.1) |  |
| No | 344 (83.1) | 342 (82.6) |  | 1,276 (43.5) | 1,258 (42.9) |  |
| NAFLD |  |  | 0.000 |  |  | 0.026 |
| Yes | 3 (0.7) | 3 (0.7) |  | 1,084 (36.9) | 1,048 (35.7) |  |
| No | 411 (99.3) | 411 (99.3) |  | 1,850 (63.1) | 1,886 (64.3) |  |
| Encephalopathy |  |  | 0.000 |  |  | 0.033 |
| Yes | 1 (0.2) | 1 (0.2) |  | 24 (0.8) | 16 (0.6) |  |
| No | 413 (99.8) | 413 (99.8) |  | 2,910 (99.2) | 2,918 (99.5) |  |
| Lung diseases |  |  | 0.069 |  |  | 0.046 |
| Yes | 30 (7.3) | 23 (5.6) |  | 1,080 (36.8) | 1,016 (34.6) |  |
| No | 384 (92.8) | 391 (94.4) |  | 1,854 (63.2) | 1,918 (65.4) |  |
| ARB  Yes  No | 96 (23.2)  318 (76.8) | 110 (26.6)  304 (73.4) | 078 | 620 (21.1)  2,314 (78.9) | 404 (13.8)  2,530 (86.2) | 0.195 |
| ICS  Yes  No | 39 (9.4)  375 (90.6) | 27 (6.5)  387 (93.5) | 0.107 | 126 (4.3)  2,808 (95.7) | 102 (3.5)  2,832 (96.5) | 0.042 |
| OCS  Yes  No | 84 (20.3)  330 (79.7) | 97 (23.4)  317 (76.6) | 0.076 | 906 (30.9)  2,028 (69.1) | 804 (27.4)  2,130 (72.6) | 0.077 |
| Vaccine  Yes  No |  |  |  | 296 (10.1)  2,638 (89.9) | 607 (20.7)  2,326 (79.3) | 0.298 |
| CCI (mean, SD) | 0.93 (1.34) | 0.92 (1.28) | 0.013 | 1.78 (2.77) | 1.46 (2.51) | 0.122 |

Abbreviations: NHIS, the national health insurance service; JBUH, Jeonbuk national university hospital; CDM, common data model; UDCA, ursodeoxycholic acid; SMD, standardized mean difference; DM, diabetes mellitus; NAFLD, nonalcoholic fatty liver disease; ARB, Angiotensin receptor blocker; ICS; Inhaled corticosteroid; OCS, Oral corticosteroid; CCI, Charlson comorbidity index; SD, standard deviation.

**Table S3.** Incidence per 10000 person-years and hazard ratios for COVID-19 infection in the NHIS and JBUH CDM susceptibility study.

|  |  | | | **JBUH CDM** | | |  |  | | | **NHIS** | | | |  |
| --- | --- | --- | --- | --- | --- | --- | --- | --- | --- | --- | --- | --- | --- | --- | --- |
| Variable | Total | Cases | inci10000 | Adjusted  hazard ratio | *P*-value | Unadjusted  hazard ratio | *P*-value | Total | Cases | inci10000 | | Adjusted  hazard ratio | *P*-value | Unadjusted  hazard ratio | *P*-value |
| Total | 40,592 | 153 |  |  |  |  |  | 413,226 | 18,374 |  | |  |  |  |  |
| UDCA |  |  |  |  |  |  |  |  |  |  | |  |  |  |  |
| No | 20,296 | 90 | 70.95 | Reference |  | Reference |  | 206,613 | 9,555 | 1211.47 | | Reference |  | Reference |  |
| Yes | 20,296 | 63 | 50.05 | 0.71 (0.52-0.98) | .031 | 0.71 (0.51-0.98) | .043 | 206,613 | 8,819 | 1116.83 | | 0.93 (0.90-0.96) | <.001 | 0.92 (0.89-0.95) | <.001 |
| UDCA Dose |  |  |  |  |  |  |  |  |  |  | |  |  |  |  |
| No | 20,296 | 90 | 70.95 | Reference |  | Reference |  | 206,613 | 9,555 | 1211.47 | | Reference |  | Reference |  |
| 300mg or less | 11390 | 35 | 54.09 | 0.78 (0.53-1.16) | .208 | 0.79 (0.53-1.16) | .247 | 157876 | 6608 | 1110.75 | | 0.94 (0.91-0.97) | <.001 | 0.92 (0.89-0.95) | <.001 |
| More than 300mg | 8906 | 28 | 45.77 | 0.64 (0.42-0.98) | .038 | 0.63 (0.41-0.96) | .035 | 48737 | 2211 | 1076.10 | | 0.91 (0.86-0.95) | .001 | 0.92 (0.88-0.96) | <.001 |
| Sex |  |  |  |  |  |  |  |  |  |  | |  |  |  |  |
| Female | 16,810 | 53 | 53.70 | Reference |  | Reference |  | 147,888 | 6,732 | 1272.06 | | Reference |  | Reference |  |
| Male | 23,782 | 100 | 64.92 | 1.26 (0.90-1.78) | .178 | 1.17 (0.84-1.63) | .353 | 265,338 | 11,642 | 1109.67 | | 0.81 (0.79-0.84) | <.001 | 0.84 (0.81-0.86) | <.001 |
| Age |  |  |  |  |  |  |  |  |  |  | |  |  |  |  |
| ~ 29 | 2,217 | 5 | 75.75 | 2.29 (0.89-5.88) | .086 | 2.09 (0.82-5.35) | .123 | 11,496 | 424 | 1547.59 | | 1.33 (1.20-1.47) | <.001 | 1.49 (1.35-1.64) | <.001 |
| 30 ~ 59 | 14,676 | 38 | 44.60 | Reference |  | Reference |  | 204,212 | 8,617 | 1226.42 | | Reference |  | Reference |  |
| 60 ~ 79 | 18,856 | 76 | 58.60 | 1.20 (0.81-1.77) | .363 | 1.27 (0.86-1.88) | .230 | 181,048 | 8,614 | 1105.71 | | 0.83 (0.81-0.86) | <.001 | 0.82 (0.79-0.84) | <.001 |
| 80 ~ | 4,843 | 34 | 108.87 | 2.27 (1.41-3.66) | .001 | 2.44 (1.53-3.87) | <.001 | 16,470 | 719 | 1037.46 | | 0.79 (0.74-0.86) | <.001 | 0.77 (0.71-0.83) | <.001 |
| Economic status |  |  |  |  |  |  |  |  |  |  | |  |  |  |  |
| High | - | - | - | - |  | - |  | 134,701 | 5,922 | 1133.66 | | 0.92 (0.89-0.95) | <.001 | 0.91 (0.88-0.94) | <.001 |
| Middle | - | - | - | - |  | - |  | 197,178 | 8,921 | 1221.00 | | Reference |  | Reference |  |
| Low | - | - | - | - |  | - |  | 81,347 | 3,531 | 1085.30 | | 0.87 (0.84-0.91) | <.001 | 0.86 (0.83-0.90) | <.001 |
| Residential area |  |  |  |  |  |  |  |  |  |  | |  |  |  |  |
| Metro | - | - | - | - |  | - |  | 177,862 | 9,755 | 1424.25 | | 1.49 (1.44-1.53) | <.001 | 1.48 (1.43-1.52) | <.001 |
| Rural | - | - | - | - |  | - |  | 235,364 | 8,619 | 964.70 | | Reference |  | Reference |  |
| BMI |  |  |  |  |  |  |  |  |  |  | |  |  |  |  |
| Normal | - | - | - | - |  | - |  | 187,062 | 7,844 | 1177.09 | | Reference |  | Reference |  |
| 25 ~ 30 | - | - | - | - |  | - |  | 175,275 | 8,290 | 1186.90 | | 1.01 (0.98-1.04) | .518 | 0.98 (0.95-1.01) | .203 |
| Over 30 | - | - | - | - |  | - |  | 50,889 | 2,240 | 1049.14 | | 0.83 (0.79-0.87) | <.001 | 0.85 (0.81-0.89) | <.001 |
| Ascites |  |  |  |  |  |  |  |  |  |  | |  |  |  |  |
| No | 39,863 | 147 | 60.44 | Reference |  | Reference |  | 408,437 | 18,228 | 1174.67 | | Reference |  | Reference |  |
| Yes | 729 | 6 | 63.11 | 0.90 (0.39-2.06) | .805 | 0.90 (0.40-2.04) | .799 | 4,789 | 146 | 548.86 | | 0.43 (0.37-0.51) | <.001 | 0.41 (0.35-0.48) | <.001 |
| Autoimmune  Diseases |  |  |  |  |  |  |  |  |  |  | |  |  |  |  |
| No | 39,950 | 149 | 60.06 | Reference |  | Reference |  | 342,677 | 15,170 | 1174.30 | | Reference |  | Reference |  |
| Yes | 642 | 4 | 86.33 | 1.51 (0.55-4.13) | .424 | 1.37 (0.51-3.71) | .532 | 70,549 | 3,204 | 1118.20 | | 0.98 (0.94-1.02) | .342 | 0.93 (0.89-0.96) | .001 |
| DM |  |  |  |  |  |  |  |  |  |  | |  |  |  |  |
| No | 36,185 | 122 | 58.08 | Reference |  | Reference |  | 252,492 | 10,761 | 1319.23 | | Reference |  | Reference |  |
| Yes | 4,407 | 31 | 72.67 | 1.15 (0.77-1.72) | .495 | 1.11 (0.75-1.65) | .602 | 160,734 | 7,613 | 998.22 | | 0.71 (0.69-0.73) | <.001 | 0.65 (0.63-0.67) | <.001 |
| NAFLD |  |  |  |  |  |  |  |  |  |  | |  |  |  |  |
| No | 39,179 | 150 | 63.63 | Reference |  | Reference |  | 286,942 | 12,646 | 1247.27 | | Reference |  | Reference |  |
| Yes | 1,413 | 3 | 17.65 | 0.26 (0.08-0.81) | .025 | 0.24 (0.08-0.74) | .011 | 126,284 | 5,728 | 1014.76 | | 0.77 (0.75-0.80) | <.001 | 0.75 (0.72-0.77) | <.001 |
| Encephalopathy |  |  |  |  |  |  |  |  |  |  | |  |  |  |  |
| No | 40,473 | 152 | 60.47 | Reference |  | Reference |  | 411,051 | 18,277 | 1164.99 | | Reference |  | Reference |  |
| Yes | 119 | 1 | 74.06 | 1.09 (0.15-7.93) | .932 | 1.07 (0.15-7.66) | .946 | 2,175 | 97 | 1020.02 | | 0.98 (0.80-1.20) | .845 | 0.83 (0.68-1.01) | .067 |
| Lung diseases |  |  |  |  |  |  |  |  |  |  | |  |  |  |  |
| No | 39,189 | 142 | 58.67 | Reference |  | Reference |  | 258,197 | 11,240 | 1161.51 | | Reference |  | Reference |  |
| Yes | 1,403 | 11 | 102.68 | 1.43 (0.76-2.66) | .267 | 1.65 (0.89-3.05) | .112 | 155,029 | 7,134 | 1168.26 | | 1.05 (1.02-1.08) | .001 | 0.99 (0.96-1.02) | .522 |
| ARB |  |  |  |  |  |  |  |  |  |  | |  |  |  |  |
| No | 34,325 | 111 | 54.40 | Reference |  | Reference |  | 338,634 | 16,525 | 1229.37 | | Reference |  | Reference |  |
| Yes | 6,267 | 42 | 86.31 | 1.35 (0.90-2.02) | .147 | 1.48 (1.04-2.11) | .029 | 74,592 | 3,842 | 1058.16 | | 1.04 (0.99-1.09) | .119 | 0.79 (0.76-0.82) | <.001 |
| ICS |  |  |  |  |  |  |  |  |  |  | |  |  |  |  |
| No | 38,330 | 137 | 57.88 | Reference |  | Reference |  | 392,177 | 19,276 | 1188.44 | | Reference |  | Reference |  |
| Yes | 2,262 | 16 | 99.87 | 1.41 (0.75-2.68) | .286 | 1.38 (1.00-2.81) | .050 | 21,049 | 1,091 | 1278.91 | | 1.12 (1.05-1.19) | .001 | 1.09 (1.02-1.16) | .010 |
| OCS |  |  |  |  |  |  |  |  |  |  | |  |  |  |  |
| No | 34,086 | 117 | 56.66 | Reference |  | Reference |  | 242,461 | 12,006 | 1178.76 | | Reference |  | Reference |  |
| Yes | 6,504 | 36 | 77.90 | 1.19 (0.80-1.78) | .391 | 1.31 (0.90-1.90) | .159 | 170,765 | 8,361 | 1213.97 | | 3.03 (2.88-3.20) | <.001 | 1.05 (1.02-1.05) | .010 |
| Vaccine |  |  |  |  |  |  |  |  |  |  | |  |  |  |  |
| No |  |  |  |  |  |  |  | 209,190 | 13,332 | 1512.91 | | Reference |  | Reference |  |
| Yes |  |  |  |  |  |  |  | 204,036 | 7,035 | 851.65 | | 0.16 (0.15-0.17) | <.001 | 0.57 (0.55-0.58) | <.001 |
| CCI (mean, SD) |  |  |  | 0.97 (0.81-1.16) | .741 |  |  |  |  |  | | 1.08 (1.08-1.09) | <.001 |  |  |

Abbreviations: NHIS, the national health insurance service; JBUH, Jeonbuk national university hospital; CDM, common data model; UDCA, ursodeoxycholic acid; DM, diabetes mellitus; NAFLD, nonalcoholic fatty liver disease; ARB, Angiotensin receptor blocker; ICS; Inhaled corticosteroid; OCS, Oral corticosteroid; CCI, Charlson comorbidity index; SD, standard deviation.

**Table S4.** Incidence per 10000 person-years and hazard ratios for severe COVID-19 in the NHIS and JBUH CDM severity study.

|  |  | | | | **JBUH CDM** | | |  |  | | **NHIS** | | | | |  |
| --- | --- | --- | --- | --- | --- | --- | --- | --- | --- | --- | --- | --- | --- | --- | --- | --- |
| Variable | Total | Cases | inci10000 | Adjusted  hazard ratio | | *P*-value | Unadjusted  hazard ratio | *P*-value | Total | Cases | | inci10000 | Adjusted  hazard ratio | *P*-value | Unadjusted  hazard ratio | *P*-value |
| Total | 828 | 41 |  |  | |  |  |  | 5,868 | 366 | |  |  |  |  |  |
| UDCA |  |  |  |  | |  |  |  |  |  | |  |  |  |  |  |
| No | 414 | 33 | 3417.80 | Reference | |  | Reference |  | 2,934 | 202 | | 2917 | Reference |  | Reference |  |
| Yes | 414 | 8 | 778.63 | 0.21 (0.09-0.46) | | <.001 | 0.23 (0.11-0.51) | <.001 | 2,934 | 164 | | 2333.94 | 0.77 (0.62-0.95) | .018 | 0.80 (0.65-0.99) | .035 |
| UDCA Dose |  |  |  |  | |  |  |  |  |  | |  |  |  |  |  |
| No | 414 | 33 | 3417.80 | Reference | |  | Reference |  | 2,934 | 202 | | 2917 | Reference |  | Reference |  |
| 300mg or less | 271 | 4 | 593.45 | 0.17 (0.06-0.49) | | .001 | 0.18 (0.06-0.50) |  | 2,146 | 116 | | 2256.11 | 0.73 (0.58-0.92) | .007 | 0.78 (0.62-0.97) | .034 |
| More than 300mg | 143 | 4 | 1131.78 | 0.26 (0.13-0.83) | | <.001 | 0.34 (0.12-0.96) | .042 | 788 | 48 | | 2546.22 | 0.89 (0.65-1.23) | .467 | 0.88 (0.64-1.20) | .431 |
| Sex |  |  |  |  | |  |  |  |  |  | |  |  |  |  |  |
| Female | 299 | 10 | 1370.53 | Reference | |  | Reference |  | 1,906 | 92 | | 1998.6 | Reference |  | Reference |  |
| Male | 529 | 31 | 2453.81 | 1.59 (0.77-3.28) | | .210 | 1.77 (0.87-3.61) | .115 | 3,962 | 274 | | 2930.97 | 1.65 (1.29-2.11) | <.001 | 1.45 (1.14-1.83) | .003 |
| Age |  |  |  |  | |  |  |  |  |  | |  |  |  |  |  |
| ~ 29 | 40 | 0 | 0.00 | 4.13 (0.00-Inf) | |  | - |  | 136 | 2 | | 591.96 | 0.40 (0.10-1.63) | .195 | 0.32 (0.08-1.27) | .107 |
| 30 ~ 59 | 231 | 11 | 1970.75 | Reference | |  | Reference |  | 3,100 | 142 | | 1900.26 | Reference |  | Reference |  |
| 60 ~ 79 | 376 | 23 | 2574.44 | 1.26 (0.60-2.64) | | .542 | - |  | 2,340 | 172 | | 3124.72 | 1.61 (1.29-2.02) | <.001 | 1.63 (1.30-2.03) | <.001 |
| 80 ~ | 181 | 7 | 1588.73 | 0.70 (0.26-1.90) | | .480 | - |  | 292 | 50 | | 7853.52 | 3.56 (2.51-5.04) | <.001 | 3.91 (2.84-5.40) | <.001 |
| Economic status |  |  |  |  | |  |  |  |  |  | |  |  |  |  |  |
| High | - | - | - | - | |  | - |  | 1,916 | 128 | | 2823.85 | 1.29 (1.01-1.65) | .041 | 1.34 (1.05-1.71) | .019 |
| Middle | - | - | - | - | |  | - |  | 2,588 | 130 | | 2090.18 | Reference |  | Reference |  |
| Low | - | - | - | - | |  | - |  | 1,364 | 108 | | 3375.75 | 1.41 (1.09-1.83) | .009 | 1.60 (1.24-2.06) | <.001 |
| Residential area |  |  |  |  | |  |  |  |  |  | |  |  |  |  |  |
| Rural | - | - | - | - | |  | - |  | 2,840 | 176 | | 2603.09 | Reference |  | Reference |  |
| Metro | - | - | - | - | |  | - |  | 3,028 | 190 | | 2642.39 | 1.03 (0.84-1.26) | .776 | 1.01 (0.83-1.25) | .921 |
| Ascites |  |  |  |  | |  |  |  |  |  | |  |  |  |  |  |
| No | 814 | 39 | 1987.35 | Reference | |  | Reference |  | 5,786 | 350 | | 2540.29 | Reference |  | Reference |  |
| Yes | 14 | 2 | 6541.22 | 5.42 (1.24-23.77) | | .025 | 3.12 (0.75-12.92) | .118 | 82 | 16 | | 9211.36 | 2.46 (1.43-4.22) | .001 | 3.34 (2.03-5.52) | <.001 |
| Autoimmune  Diseases |  |  |  |  | |  |  |  |  |  | |  |  |  |  |  |
| No | 808 | 40 | 2056.54 | Reference | |  | Reference |  | 4,844 | 306 | | 2658.3 | Reference |  | Reference |  |
| Yes | 20 | 1 | 2084.52 | 1.18 (0.16-8.77) | | .871 | 1.00 (0.14-7.27) | 1.000 | 1,024 | 60 | | 2458.46 | 0.93 (0.70-1.22) | .617 | 0.93 (0.70-1.23) | .617 |
| DM |  |  |  |  | |  |  |  |  |  | |  |  |  |  |  |
| No | 686 | 33 | 1996.22 | Reference | |  | Reference |  | 2,534 | 114 | | 1859.81 | Reference |  | Reference |  |
| Yes | 142 | 8 | 2353.89 | 0.82 (0.36-1.85) | | .637 | 1.17 (0.54-2.53) | .691 | 3,334 | 252 | | 3221.67 | 1.44 (1.15-1.81) | .002 | 1.71 (1.37-2.14) | <.001 |
| NAFLD |  |  |  |  | |  |  |  |  |  | |  |  |  |  |  |
| No | 822 | 40 | 2022.22 | Reference | |  | Reference |  | 3,736 | 254 | | 2872.68 | Reference |  | Reference |  |
| Yes | 6 | 1 | 6684.98 | 2.58 (0.32-20.93) | | .374 | 3.34 (0.46-24.30) | .233 | 2,132 | 112 | | 2191.89 | 0.81 (0.65-1.02) | .061 | 0.77 (0.61-0.96) | .028 |
| Encephalopathy |  |  |  |  | |  |  |  |  |  | |  |  |  |  |  |
| No | 826 | 41 | 2062.43 | Reference | |  | Reference |  | 5,828 | 358 | | 2581.84 | Reference |  | Reference |  |
| Yes | 2 | 0 | 0.00 | 4.12 (0.00-Inf) | |  |  |  | 40 | 8 | | 9346.99 | 1.40 (0.66-2.97) | .381 | 3.23 (1.60-6.51) | .001 |
| Lung diseases |  |  |  |  | |  |  |  |  |  | |  |  |  |  |  |
| No | 775 | 36 | 1924.18 | Reference | |  | Reference |  | 3,772 | 222 | | 2467.19 | Reference |  | Reference |  |
| Yes | 53 | 5 | 4096.52 | 2.43 (0.88-6.76) | | .087 | 2.09 (0.82-5.33) | .123 | 2,096 | 144 | | 2906.98 | 1.08 (0.87-1.34) | .485 | 1.18 (0.96-1.46) | .116 |
| ARB |  |  |  |  | |  |  |  |  |  | |  |  |  |  |  |
| No | 622 | 29 | 1933.76 | Reference | |  | Reference |  | 4,844 | 292 | | 2529.00 | Reference |  | Reference |  |
| Yes | 206 | 12 | 2432.52 | 0.96 (0.42-2.24) | | .923 | 1.25 (0.64-2.45) | .514 | 1,024 | 52 | | 2117.17 | 1.08 (0.72-1.62) | .710 | 0.84 (0.62-1.12) | .261 |
| ICS |  |  |  |  | |  |  |  |  |  | |  |  |  |  |  |
| No | 762 | 36 | 1958.48 | Reference | |  | Reference |  | 5,640 | 330 | | 2451.48 | Reference |  | Reference |  |
| Yes | 66 | 5 | 3229.52 | 0.92 (0.24-3.57) | | .903 | 1.63(0.64-4.16) | .306 | 228 | 14 | | 2588.39 | 1.36 (0.75-2.46) | .311 | 1.05 (0.62-1.80) | .86 |
| OCS |  |  |  |  | |  |  |  |  |  | |  |  |  |  |  |
| No | 647 | 28 | 1789.84 | Reference | |  | Reference |  | 4158 | 274 | | 2780.17 | Reference |  | Reference |  |
| Yes | 181 | 13 | 3033.11 | 1.40 (0.66-2.97) | | .381 | 1.66 (0.86-3.21 | .131 | 1710 | 70 | | 1688.12 | 0.70 (0.47-1.06) | .079 | 0.61 (0.47-0.80) | .002 |
| Vaccine |  |  |  |  | |  |  |  |  |  | |  |  |  |  |  |
| No |  |  |  |  | |  |  |  | 4,964 | 326 | | 2771.80 | Reference |  | Reference |  |
| Yes |  |  |  |  | |  |  |  | 904 | 18 | | 803.28 | 0.23 (0.14-0.39) | <.001 | 0.30 (0.18-0.47) | <.001 |
| CCI (mean, SD) |  |  |  | 1.06 (0.72-1.56) | | .768 |  |  |  |  | |  | 1.07 (1.01-1.14) | .022 |  |  |

Abbreviations: NHIS, the national health insurance service; JBUH, Jeonbuk national university hospital; CDM, common data model; UDCA, ursodeoxycholic acid; DM, diabetes mellitus; NAFLD, nonalcoholic fatty liver disease; ARB, Angiotensin receptor blocker; ICS; Inhaled corticosteroid; OCS, Oral corticosteroid; CCI, Charlson comorbidity index; SD, standard deviation.
